# Health Insurance Notes

**Published:** 1923-08

**Authors:** 


### NATIONAL HEALTH INSURANCE NOTES.

### THE GOOD NAME OF THE SERVICE.

AT the June Conference of the Panel Committees, a resolution was passed expressing the opinion of the Conference that the regulations with regard to complaints should be tightened in such a way as to ensure the removal from the panel of any practitioner who, by constant indifference to his duties, endangered the good name of the service. This is another welcome sign of the fact that the doctors as a body are jealous for their reputation. They realise, as well as anybody, that there are cases not sufficiently bad in an individual instance to justify removal from the panel, where the cumulative effect of complaints of indifference or worse leaves no doubt in the minds of reasonable men that the service is being prejudiced by the continuance of the offender on the panel. If the doctors' representatives will now, in conference with the Ministry of Health, arrive at some practical means of giving effect to their resolution, they will have provided the Consultative Council with one answer to their request, to which we referred last month, that something must be done in this matter.

### THE APATHY OF THE INSURED.

It is well that we should remind ourselves from time to time that the income of the panel practitioner depends upon the number of those who select him as their doctor. It is therefore to be hoped that, if the insured patients are given the right to change their doctors at any time, as the Panel Conference have recommended, they will shake off some of their apathy and leave the doctor who shows "constant indifference to his duties" in such numbers that the effect on his quarter's cheque will give him "furiously to think." When there is a tooth in the head capable of doing good work but inclined to rot, it is better to stop it in the first instance; if it goes on rotting, it must come out.

### NO NEED FOR CANT.

Dr. Harry Roberts, whose book on *A National Health Policy* we review elsewhere, speaks plainly on a good many subjects. In regard to slum practice he says that, in general, a doctor will not live and work in a slum area for such fees as he might expect to receive from those who dwell there when he has the chance of earning good fees in a more pleasant environment. There is no need, he says, to talk cant. "I well remember," Dr. Roberts writes, of the days before there were panels, "the daily embarrassment that one experienced on discovering that, in order to pay even one's paltry fee, some article of the barest necessity had to be pawned. . . . So far as the insured population goes, the Panel system has at least put an end to that horror." The scrap-the-lot critics might read this and, in connection with the same subject, the statement made at the Panel Conference that, in the Cowgate and Canongate districts of Edinburgh, before the Insurance Act came into operation, there was only one doctor, and he was only on the fringe of the area, and that now six doctors practised in the district; also the reminder, in a recent article in the *Leeds Mercury*, that the old Friendly Societies used to select their "lives" rather carefully, and that rarely, if ever, did they provide a medical service for women.

### HEALTHY INSURANCE FUNDS.

Notwithstanding the admission of practically the whole employed population to the benefits of the Health Insurance scheme, it is clear from the document in which the British Medical Association review the financial position of the National Health Insurance Funds that the Funds themselves are in a healthy condition. We are reminded that the Valuation of Approved Societies up to 1918 showed a total surplus of £23,500,000, and that the continued low sickness experience since that date gives a strong indication of further large profits subsequently. Further, that behind the profits already made there are substantial balances in various subsidiary accounts of the main Fund, making its position additionally secure. The most arresting statement in the document, from the taxpayers' point of view, is that in which it is pointed out that the Societies have made these profits over periods when large special grants have been received from the Exchequer on the assumption—which experience of the working of the Act has not yet supported—that the Insurance funds were inadequate to meet the normal and necessary liabilities. It is, at any rate, comforting to know that these raids on the Exchequer have come to an end. So far as the doctors are concerned, the moral of the document is understood to be that if they make out a good case for the payment of a capitation of a certain amount, it will be of no use to tell them that there are no funds available to pay them so large an amount.

### IRISH HEALTH INSURANCE.

There is something essentially Irish about a scheme of Health Insurance which does not include Medical Benefit. The only reason for this omission in the Irish scheme has always appeared to be that they simply had to be different from those accursed people in Great Britain. And now, just as the Dail are making the position clear, by their Insurance Bill, that they have no connection with the firms over the border and across the Channel, we read that the North of Ireland is awakening to the need for Medical Benefit, in spite of the hard things which are said about it over here. But then, of course, in Ireland they say that they are accustomed sometimes to think that we do not mean what we say.

### WITHOUT HOPE.

The General Federation of Trade Unions have published a report on the Sickness Conditions in Dundee. There are some strong expressions in the report, which makes very ugly reading. When, therefore, the Society found "hopeless folk existing under conditions which perpetuate hopelessness," they turned to section 63 of the National Insurance Act, 1911, to see what action they could take. The Controller of National Health Insurance has recently invited Societies to explore the possibilities of this section from which, in 1911, so much was hoped. Here is a Society which says that the section is useless. If that is a fair statement, the section should be repealed—or made workable.

